# Iranian Emotional Experience and Expression During the COVID-19 Crisis

**DOI:** 10.1177/1010539520937097

**Published:** 2020-07

**Authors:** Khodabakhsh Ahmadi, Mohammad Arash Ramezani

**Affiliations:** 1 Behavioral Sciences Research Center, Baqiyatallah University of Medical Sciences, Tehran, Iran; 2 Iranian Institute of Emotion-Focused Therapy, Tehran, Iran

The outbreak of coronavirus disease 2019 (COVID-19) was first reported in Wuhan, China in December 2019.^1^ This disease has spread rapidly in many countries, including Iran, where a cumulative number of cases has been reported since the end of February 2020.^2^ From the beginning of this pandemic, all public places, even mosques and holy shrines, were closed in Iran, and social distancing and home quarantine were encouraged. A remarkable point about the spread of this disease in Iran was the coincidence of the COVID-19 pandemic with the Iranian New Year (“Nowruz”). Consequently, the New Year ceremonies, including family visits, long-distance travel, celebrations, and gatherings, were suspended.

In the COVID-19 pandemic, people’s mental health has been negatively affected due to the increase in morbidity and mortality, besides changes in living conditions and self-quarantine. Almost everyone is experiencing a type of mental health problem, such as anxiety, depression,^3^ fear of death, anger, posttraumatic stress disorder,^4^ and suicide.^5^ However, the associated awareness of potential health-related threat can be protective and prompt health-promoting behavior, when if excessive can be detrimental.^6^

From an Emotion-Focused Therapy perspective, the COVID-19 pandemic can trigger maladaptive emotional schemas, as people tend to suppress their emotions and rely on avoidance mechanisms.^7^ In other words, many people may ignore this real-life problem and dismiss the COVID-19 outbreak. In Iran, it took about 1 month for people to accept the life-threatening nature of COVID-19; even some politicians failed to acknowledge the importance of this disease. Denial is the first stage of emotional expression in critical situations. Therefore, people may misinterpret and catastrophize their thoughts, behaviors, and bodily sensations regarding the COVID-19 pandemic. Reactions to this critical condition depend on the emotional processing style. In this article, we tried to observe and report the Iranians’ emotional processing pattern since the beginning of the COVID-19 pandemic.

Fear has been the most important and common emotion during the COVID-19 pandemic. This primary adaptive emotion has encouraged people to maintain sanitation during quarantine. Iranian people, similar to other populations, may experience fear as an adaptive emotion.^8^ However, if fear is experienced maladaptively, secondary emotions, such as anxiety, worry, panic, and phobia, may appear. People during quarantine may become very anxious and phobic due to social isolation, especially when they are unable to understand their anxiety. The COVID-19 pandemic has created catastrophic expectations about the future and has caused major anxiety in people. There are numerous discussions and misconceptions about this disease in the cyberspace and social media, triggering maladaptive emotions, such as fear, among people.

Shame caused getting virus, as an adaptive emotion, can protect one’s privacy. During the COVID-19 crisis, this shame has encouraged people to social distancing. Due to the common purpose and unity of Iranian people in eradicating COVID-19, they have experienced shame adaptively; only some groups, who have lost their jobs due to the pandemic, feel worthless.

Also, in the COVID-19 pandemic, anger has been triggered in the following three situations:

1. Value conflicts: Due to the Corona crisis, mosques and shrines have been closed; therefore, people who used to pray in these sites cannot visit them anymore.
2. The coincidence of the peak of the COVID-19 pandemic with the New Year holidays (Nowruz) in Iran: Nowruz provides this opportunity for people to go sightseeing, socialize with others, and travel; however, the pandemic prevented these holiday activities.
3. Threatening of the family status: Since family members are required to stay at home and avoid visiting their relatives and friends, family conflict may arise. Also, couples who did not have any major conflicts before the outbreak may be involved in new conflicts.^9^

Moreover, some people feel grief and sadness due to the loss of their loved ones to COVID-19 or other causes. In the Iranian culture, burial and mourning ceremonies are religious rituals, which can soothe people and accelerate the grieving process. Due to social restrictions and quarantine during this pandemic, people cannot attend the mourning ceremonies, which are special events in the Islamic and Iranian culture. Consequently, they may feel unresolved grief and cannot fully express their sadness. Overall, delayed grief, grief disorder, and subsequent depression symptoms are predictable.

The COVID-19 pandemic has imposed emotional burdens on the public. It seems that after the end of this pandemic, another pandemic of mental and social disorders will arise. Generally, the majority of community interventions are based on cognitive-behavioral models.^10^ We suggest that emotional problems and related treatments be also included in these interventions. In addition to Emotion-Focused Therapy, patience is also of paramount importance in stressful situations. In religious terms, patience results from the individual’s trust in a higher power and stems from his/her understanding of mental and spiritual principles; therefore, people with patience have a more secure state of mind. In other words, patience entails acceptance, positivity, responsibility, commitment, faith, hope, and trust.
